# The potential of carcinoembryonic antigen, p53, Ki-67 and glutathion Stransferase-π as clinico-histopathological markers for colorectal cancer^☆^

**DOI:** 10.1016/S1674-8301(10)60008-5

**Published:** 2010-01

**Authors:** Zhenyu He, Chuanbing Shi, Hao Wen, Fanglong Li, Baolin Wang, Jie Wang

**Affiliations:** a Departments of General Surgery and; b Department of Pathology, the Second Affiliated Hospital to Nanjing Medical University, Nanjing, Jiangsu, 210011, China.; c Interventional Radiology, the First Affiliated Hospital, Nanjing Medical University, Nanjing, Jiangsu, 210011, China.; d The First Clinic College , Nanjing Medical University, Nanjing, Jiangsu, 210029, China

**Keywords:** colorectal cancer, carcinoembryonic antigen, p53, Ki-67, glutathion stransferase-π, lymph node metastasis

## Abstract

**Objective:**

Colorectal cancer is one of the major contributors to cancer death worldwide. Lack of reliable colorectal cancer markers has hampered the management of these cancer patients. Our main purpose was to study the correlation between histopathological variables of colorectal adenocarcinomas and identify histopathological markers that are of prognostic value in patients with colorectal cancer.

**Methods:**

In the present study, we examined the expression of carcinoembryonic antigen (CEA), p53, Ki-67 and glutathion Stransferase (GST) -π by using immunohistochemical staining methods in 126 colorectal carcinoma patients and evaluated the lymph node metastasis status in these patients by histopathological examination.

**Results:**

The positive rates of CEA, p53, Ki-67 and GST-π expression in the colorectal cancer tissue specimens examined were 95.23%, 55.56%, 53.38% and 82.30%, respectively. Expression of p53 and Ki-67 was significantly correlated with the Dukes stages of the tumor, with higher levels of these proteins in Dukes'C and D tumors than those in Dukes' A and B tumors. Furthermore, the expression of p53, GST-π and Ki-67 correlated with prognosis of patients with colorectal cancer. Additionally, the expression of p53 in colorectal cancer was closely related to the expression of Ki-67 and the expression of GST-π was directly correlated with that of p53.

**Conclusion:**

The expression of CEA, p53, Ki-67 and GST-π was correlated with various clinical features of patients with colorectal cancer. The combined use of these histopathological markers appeared to be a promising tool in predicting the prognosis of patients with this type of cancer.

## INTRODUTION

Colorectal cancer is one of the leading causes of cancer death in China and worldwide[1]. Surgery is the mainstay of therapy for those with localized disease at the time of diagnosis and adjuvant chemotherapy is offered to those patients with lymph node metastasis. Locoregional recurrence of the tumor after curative resection still remains problematic. Up to now, the only reliable predictor of prognosis for patients with colorectal cancer is tumor staging by the Dukes system[2]. It is therefore important to identify other potential clinico-histopathological markers that can predict the aggressive behavior of the tumor and also the overall survival of patients with this type of cancer.

Several biological markers such as the carcinoembryonic antigen (CEA), the multifunctional protein (p53), the marker of cellular proliferation (Ki-67), and the placental form of glutathione Stransferase (GST-π), have been shown to be related to the growth and infiltration, recurrence and metastasis, cell proliferation activities and prognosis of colorectal cancer[3],[4],[5],[6]. However, there still exists much controversy as to the validity and efficacy of these biological markers in serving as a predictor of the overall prognosis of patients with colorectal cancer[7]. In the current investigation, we utilized immunohistochemical methods to detect the expression of CEA, p53, Ki-67 and GST-π proteins in colorectal carcinoma tissues, and further examined the expression of these proteins for their correlation with several clinico-histopathological parameters, including gender, age, tumor size, degree of tumor differentiation, Dukes stages, lymph node metastasis and patients' prognosis. Our findings indicate these histopathological markers may be of value in predicting the aggressive behavior of the tumor and possibly the prognosis of patients with colorectal cancer.

## MATERIALS AND METHODS

### Patients

The case series in our investigation consisted of 126 patients diagnosed with colorectal cancer who were admitted into the Second Affiliated Hospital of Nanjing Medical University, Nanjing, Jiangsu, China from January 2000 to June 2007. Of these cases, 74 were male and 52 female; their age ranged from 34 to 85 years old with a mean age of 63.79±14.5 years. Tumors were classified according to the Dukes system as follows: Dukes stage A, *n* = 22; B, *n* = 62; C, *n* = 30; and D, *n* = 12. Fifty-four tumors were located on the right side and 42 on the left side of the colon, and 34 were in the rectum. Patient time experience started at their operation date and ended at the time of their death or at the conclusion of the study. All patients received primary surgical therapy. Thirty-one patients received radiotherapy and 84 patients were treated with chemotherapy and 9 patients received both radiotherapy and chemotherapy, whereas 20 patients received no additional chemotherapy.

These patients were followed up for local recurrence, metastasis and survival for a period of 12 to 96 months with a median follow-up of 36 months. Twenty patients died during follow-up and 14 patients had evidence of recurrence. Of the 14 patients with recurrent disease, 9 received re-operation.

Written informed consent for clinical treatment and use of resected specimens was obtained from all patients upon admission in accordance with the guidelines set by the hospital ethics review committee. The current study was approved by the committee.

### Immunohistochemical determinations

Tumor samples were collected at the time of operation. With the removal of necrotic tissues, tumor tissue blocks of 2 cm×2 cm×0.5 cm were collected and fixed in 10% neutral formalin solution for 24 h and subsequently subjected to conventional paraffin embedding. Histological paraffin sections, 4 µm thick, were prepared by standard method, stained with hematoxylin and eosin, and examined by light microscopy. Serial sections from respective specimens containing lesions of interest were used for the immunohistochemical staining of the following proteins, CEA, p53, Ki-67 and GST-π (primary antibodies obtained from Santa Cruz, CA, USA). Biotin-labeled secondary antibody and streptavidin-peroxidase complex were purchased from Maixin Co. (Fujian, China). Tissue sections were treated sequentially with anti-CEA, p53, Ki-67 or GST-π antibody, biotin-labeled secondary antibody and streptavidin-peroxidase complex. The peroxidase binding sites were detected using diaminobenzidine (DAB) as the substrate. Negative controls were obtained by omission of the primary antibodies.

Positive staining was exclusively nuclear for p53 and Ki-67 and cytoplasmic for CEA and GST-π. Quantification was done at the microscope by two independent observers scoring a total of 500 tumor cells from consecutive areas of the same tumor. Positivity was expressed as the percentage of the stained cells in the total number of counted tumor cells. The value of 5% was used as cutoff values to define negative tumors in clinical analysis (-); the values between 5% and 30% were considered positive (+); and those above 30% strongly positive (++).

### Statistical analysis

All statistical analyses were carried out using the SPSS statistical software (Version 16.0, SPSS Inc., USA). Fisher exact test was used to analyze the relationship between target protein expression and clinical and pathological characteristics. Survival curves were plotted by the Kaplan-Meier method and compared by Log-rank test. *P* < 0.05 in all cases was considered statistically significant.

## RESULTS

**Fig. 1:**
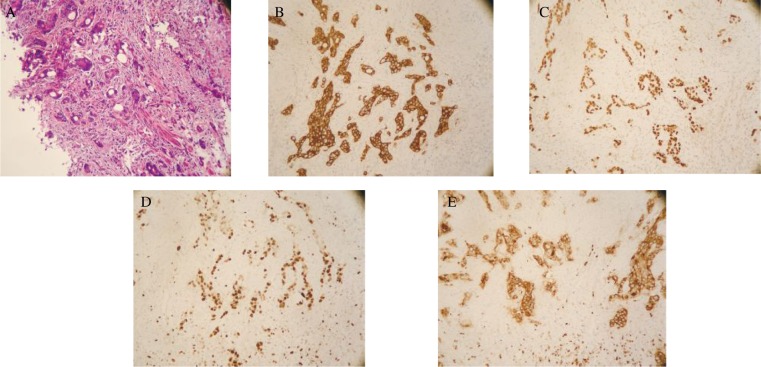
HE stain (A) and CEA(B), p53(C), Ki-67(D), GST(E) antigen stain of an adenocarcinoma of the colon specimen. A: Histological specimen shows a differentiated adenocarcinoma of the colon (Hematoxylin and eosin stain, ×200); B: Adenocarcinoma of the colon stained with anti-CEA antibody. Tumor cells display intense cytoplasmic staining of CEA (×200). C: A colorectal carcinoma specimen stained with anti-p53 antibody. Tumor cells show strong distinct nuclear staining of the protein (×200); D: An adenocarcinoma of the colon stained with anti-Ki-67 antibody displays nuclear staining of the protein (×200); E: Histological section from an adenocarcinoma of the colon specimen shows positive staining of GST- antibody in the cytoplasm (×200).

Tumor tissues in which over 5% of the component cells were stained immunohistochemically using the anti-CEA, p53, Ki-67 or GST-π antibody were evaluated as being positive for CEA, p53, Ki-67 and GST-π staining. ***Fig. 1A*** shows the hematoxylin and eosin staining of differentiated adenocarcinoma of the colon. CEA was expressed as intense cytoplasmic staining using the anti-CEA antibody ***(Fig. 1B)*** and was detected in 95.23 % (120/126) of all the tumors examined ***(Table 1)***. No statistical difference was found in the level of its expression in these patients with regards to gender or age. CEA expression also did not differ with regards to tumor size or position, degree of differentiation, or Dukes stages of the tumor. Additionally, neither lymph node metastasis status nor prognosis of these patients was correlated with the expression of CEA. The expression of CEA was directly correlated with that of p53 and GST-π (*P* = 0.017, *P* = 0.035, respectively).

**Table 1.** The relationship between the expression of CEA, p53, Ki-67 and GST-π and the clinical and pathological staging of colorectal carcinoma

Factor		CEA expression				p53 expression				Ki-67 expression				GST-π expression			
		(-)	(+)	(++)	*P*	(-)	(+)	(++)	*P*	(-)	(+)	(++)	*P*	(-)	(+)	(++)	*P*
Gender	Male	4	28	42	0.091	36	26	12	0.268	34	32	8	0.872	14	28	32	0.785
	Female	2	30	20		20	26	6		26	20	6		8	18	26	
Age	< 45	0	4	4	0.086	0	8	0	0.005	0	8	0	0.002	2	2	4	0.001
	45-60	2	24	12		22	10	6		20	10	8		12	18	8	
	> 60	4	30	46		34	34	12		40	34	6		8	26	46	
Size of the tumor	<= 5 cm	4	42	32	0.052	44	38	6	0.000	42	40	6	0.054	12	28	48	0.013
	> 5 cm	2	16	30		12	14	12		18	12	8		10	18	10	
Position of the tumor	Right hemicolon	4	20	18	0.134	20	26	4	0.280	28	18	4	0.527	6	24	20	0.042
	Left hemicolon	2	26	22		20	14	8		16	20	6		6	16	20	
	Rectum	0	12	22		16	12	6		16	14	4		10	6	18	
Differentiated degree	Relatively good	4	40	30	0.060	38	24	12	0.057	50	14	10	0.000	18	20	36	0.008
	Relatively poor	2	18	32		18	28	6		10	38	4		4	26	22	
Lymph node metastasis	Yes	2	36	26	0.062	36	36	8	0.182	44	32	4	0.005	16	30	34	0.492
	No	4	22	36		20	16	10		16	20	10		6	16	24	
Dukes staging	A,B	5	50	28	0.058	34	48	2	0.000	56	24	4	0.000	18	32	34	0.139
	C,D	1	18	24		2	4	36		4	28	10		4	14	24	
Prognosis	≥ 3 year	6	46	60	0.102	52	48	12	0.017	58	46	8	0.001	16	42	54	0.044
	< 3 year	0	10	4		4	4	6		2	6	6		6	4	4	

**Table 2.** The relationship between the expression of CEA, p53, Ki-67 and GST-π in colorectal carcinoma

		CEA			*P*	p53			*P*	Ki-67			*P*
		(++)	(+)	(-)		(++)	(+)	(-)		(++)	(+)	(-)	
p53	(++)	2	4	0	0.017								
	(+)	2	12	4									
	(-)	52	36	14									
Ki-67	(++)	2	2	2	0.054	32	10	4	0.0018				
	(+)	6	12	0		20	16	6					
	(-)	52	38	12		8	6	18					
GST-π	(++)	0	4	2	0.035	10	14	20	0.0039	10	16	34	0.99
	(+)	3	10	2		12	14	24		8	10	20	
	(-)	8	40	54		0	4	28		4	6	18	

The p53 was detected in 55.56% of all the tumors examined ***(Table 1)*** and positive tumor specimens showed strong distinct nuclear staining of the protein ***(Fig. 1C)***. The relationship between p53 expression and several clinicopathological variables is summarized in ***Table 1***. It was detected in 68.42% of the tumors greater than 5 cm in size and 50.00% of the tumors smaller than 5 cm in size (*P* < 0.05). The protein also exhibited a markedly higher rate of positivity in Dukes stage C and D tumors (90.90%) than that in Dukes stage A and B tumors (59.52%) (*P* < 0.05). Furthermore, the rate of tumors with positive p53 expression was slightly higher in those with a poor prognosis (71.43%) than that in those with a better prognosis (65.22%) (*P* < 0.05). However, no statistical difference was found in the level of its expression in patients with regards to the differentiation status or lymph node metastasis.

The cellular proliferation marker Ki-67 was mainly expressed in the nuclei ***(Fig. 1D)*** and detected in 53.38% of all the tumors examined ***(Table 1)***. The expression of Ki-67 was examined for its correlation with several clinicopathological parameters. Ki-67 was expressed at a significantly higher rate in those with Dukes stage C and D (90.48%) than those with Dukes stage A and B (33.33%) (*P* < 0.05). The protein also exhibited a markedly higher rate of positivity in relatively poorly differentiated tumors (80.76%) than in relatively well differentiated tumors (32.43%) (*P* < 0.05). Furthermore, the rate of Ki-67 expression was higher in those with a poor prognosis (85.71%) than in those with a better prognosis (48.21%) (*P* < 0.05). On the other hand, Ki-67 had a lower rate of positive expression in those with lymph node metastasis (42.11%) than in those without such metastasis (68%) (*P* < 0.05). Additionally, the expression of p53 in colorectal cancer was closely related to the expression of Ki-67 (*P* = 0.0018) ***(Table 2)***.

GST-π showed cytoplasmic staining in positive tumor specimens ***(Fig. 1E)*** and was detected in 82.3% of all the tumors examined. The expression of GST-π was further examined for its correlation with several clinicopathological variables. It was detected in 88.63% of the tumors greater than 5 cm in size and 54.17% of the tumors smaller than 5 cm in size (*P* < 0.05). The protein also exhibited a markedly higher rate of positivity in relatively poorly differentiated tumors (92.30%) than relatively well differentiated tumors (75.67%) (*P* < 0.05). Furthermore, the rate of GST-π expression was lower in those with a poor prognosis (57.14%) than that in those with a better prognosis (90.57%) (*P* < 0.05). Additionally, neither lymph node metastasis status nor Dukes stage was correlated with the expression of GST-π. Also, the expression of GST-π was directly correlated with that of p53 (*P* = 0.0039), but not correlated with the expression of Ki-67 (*P* = 0.99).

**Fig. 2:**
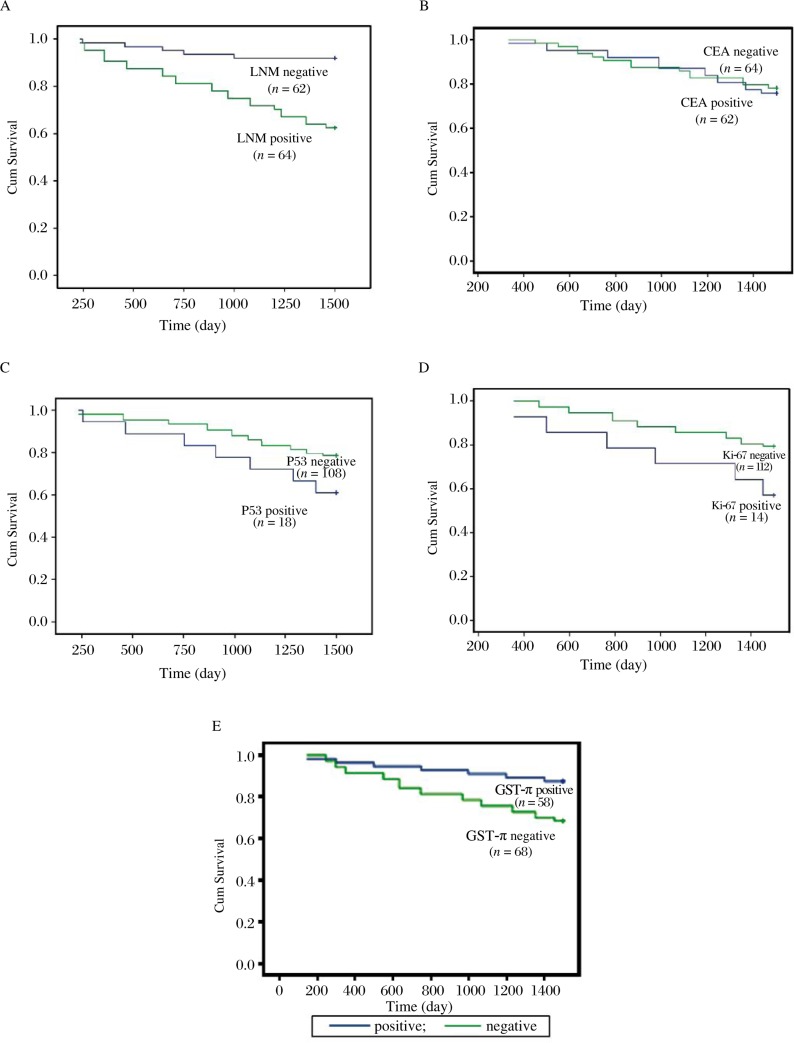
Kaplan-Meier survival curve analyzed in colorectal cancer patients according to the presence or absence of lymph node metastasis (LNM) (A, *P* = 0.0001), the negative or positive expression of CEA (B, *P* = 0.805), p53 (C, *P =* 0.091), Ki-67 (D, *P* = 0.042) and GST-π (E, *P* = 0.014).

We further calculated the survival rates in the 126 colorectal cancer patients according to the presence or absence of lymph node metastasis, and the negative or positive expression of CEA, p53, Ki-67 and GST-π ***(Fig. 2)***. The results showed that those patients without lymph node metastasis (mean survival 1 429 days; 95% CI: 1 366-1 491 days) exhibited a significantly longer survival than those with lymph node metastasis (mean survival 1 237 days; 95% CI: 1 135-1 338 days) (*P* < 0.01). There were also significant differences between the survival curves according to the expression of Ki-67 (*p* = 0.042) and GST-π (*P* = 0.014), but not according to CEA and p53 (*P* = 0.805, *P* = 0.091, respectively).

## DISCUSSION

The use of a single tumor marker results in poor specificity and low positivity rate in clinical applications[8], including colorectal cancer. Over the past two decades, many studies have been performed to study the correlation between histopathological variables of colorectal adenocarcinomas and prognosis of patients with this type of cancer[9]–[11]. Our interest was to identify histopathological markers that are of prognostic value in patients with colorectal cancer. CEA is a complex glycoprotein produced by more than 90% of colorectal cancers and contributes to the malignant characteristics of this type of cancer. CEA might serve as a predictor of survival in patients with Dukes C colorectal cancer[12]. In the current research, using conventional immunohistochemical methods, we detected the presence of CEA in 95.23% of all the tumors examined. However, when analyzed for its correlation with several clinico-histopathological parameters including gender, age, tumor size, degree of tumor differentiation, Dukes stages, lymph node metastasis or patients' prognosis, CEA expression was not correlated with any of these clinicopathological parameters. These results suggest that the role of determining CEA expression in colorectal cancer samples is rather limited in predicting the behavior of this type of cancer or the survival of patients.

The protein encoded by the p53 gene controls multiple cellular functions, including cell proliferation, DNA repair and apoptosis[13]. Though overexpression of the protooncogen p53 has been shown to be an independent predictor for survival in patients with colorectal cancer[14], it failed to be borne out by other studies[15],[16]. In our current research, we detected the presence of p53 in 55.56% of all the tumors examined, which is consistent with the expression rate of p53 reported previously for colorectal cancer[17]. We further demonstrated a higher rate of p53 expression in Dukes stage C and D tumors (90.90%) than that in Dukes stage A and B tumors (59.52%) (*P* < 0.05). We also detected higher levels of p53 expression in those patients with a poor prognosis (71.43%) than those in those patients with a better prognosis (65.22%). Our findings are consistent with other reports that overexpressed p53 is correlated with decreased survival of patients with colorectal cancer[17]–[20].

Ki-67 serves as a marker of cellular proliferation and higher levels of Ki-67 expression in colorectal cancer is considered to be associated with poorer prognosis in patients with this type of cancer[21]–[23]. Our data also revealed a significantly higher level of Ki-67 expression in those patients with Dukes stage C and D tumors than those with Dukes stage A and B tumors. Our data confirmed that higher levels of Ki-67 expression were associated with poor prognosis in patients with colorectal cancer. These results suggest that the proliferation marker Ki-67, along with other clinicopathological markers, might be useful as a predictor for prognosis in patients with colorectal cancer.

The human placental form of GST (GST-π) is frequently overexpressed in many cancers, including colorectal cancer, and may serve as a predictive marker for diagnosis and prognosis of colorectal cancer patients[24]–[26]. Increased levels of GST-π were associated with poorer prognosis in patients with colorectal cancer[25]. However, the data from our study suggested that decreased expression of the protein was associated with features indicative of poorer prognosis in patients with colorectal cancer. The poor prognosis of patients with increased expression of GST-π is mainly due to drug resistance. Our results differed from others, which needs larger sample number to confirm. Also, we found that the expression of GST-π was directly correlated with that of p53, while both of them related to poor prognosis. We supposed that the parallel expression of these two markers was mainly manifested in the patients without lymph node metastasis, while in those patients with such metastases, the expression of GST-π was low and p53 was high.

Our Kaplan-Meier analysis of the overall survival of these patients according to the presence or absence of lymph node metastases showed that those with lymph node metastases exhibited poorer survival than those without such metastases (*P* < 0.05). Additionally, the patients with positive expression of Ki-67 and negative expression of GST-π had longer survival times than those with positive expression of GST-π and negative expression of Ki-67, which indicated that the combined detection of Ki-67 and GST-π may be of great value in the prognosis of patients with colorectal cancers.

In summary, the current investigation indicates that combined detection of the expression of CEA, p53, Ki-67 and GST-π in colorectal cancer may be of value in understanding the aggressive behavior of colorectal cancer such as the growth, recurrence, and metastasis of the tumor, and may provide useful information on the prognosis of patients with this type of cancer.
